# Navigating the AI Revolution: The Case for Precise Regulation in Health Care

**DOI:** 10.2196/49989

**Published:** 2023-09-11

**Authors:** Sandeep Reddy

**Affiliations:** 1 Deakin School of Medicine Geelong Australia

**Keywords:** artificial intelligence, AI, health care, regulation, precise regulation, patient safety, AI ethics

## Abstract

Health care is undergoing a profound transformation through the integration of artificial intelligence (AI). However, the rapid integration and expansive growth of AI within health care systems present ethical and legal challenges that warrant careful consideration. In this viewpoint, the author argues that the health care domain, due to its complexity, requires specialized approaches to regulating AI. Precise regulation can provide clear guidelines for addressing these challenges, thereby ensuring ethical and legal AI implementations.

## Introduction

Artificial intelligence (AI) has already begun to transform health care in various ways [1]. AI-powered technologies are being used to diagnose diseases, develop new treatments, and improve patient care. However, the rapid growth and integration of AI in health care systems present numerous ethical and legal challenges [2]. As AI becomes more widespread, it is increasingly important to consider how it should be regulated.

There are two main approaches to regulating AI in health care: general regulation and precise regulation. General or generic regulation of AI refers to the overarching set of rules, guidelines, and policies that govern the development, deployment, and use of AI technologies across various domains and industries. These regulations are designed to address common ethical, legal, and safety concerns associated with AI, regardless of the specific application or industry. The European Union’s proposed AI Act is an example of such regulation [3]. Precise regulation, on the other hand, refers to the specific set of rules, guidelines, and policies that are tailored to govern the development, deployment, and use of AI technologies in a particular industry or sector. These regulations are designed to address the unique challenges and considerations associated with AI applications in that industry. An example would be the US Food and Drug Administration’s (FDA) AI/Machine Learning-Based Software as a Medical Device Action Plan [4].

Although some advocate for general regulation [5,6] to apply to AI across sectors, this paper advocates for the necessity of precise regulation for health care AI. By developing regulations that are specific to the health care industry, policy makers can ensure that AI is being used in an ethical and responsible manner.

## Current Regulatory Process

AI is becoming increasingly prevalent in our society, and as such, there are many legal and ethical challenges that arise [5,6]. The challenges associated with AI include fairness, effectiveness, cybersecurity, and intellectual property, among others. In recent times, governments and organizations have called for stricter AI regulations [7,8]. This has been a response in relation to the emergence and rapid growth of generative AI. In particular, there are concerns about the potential negative impact that AI could have on society if left unchecked. Many experts are calling for a more thoughtful and deliberate approach to the development and deployment of AI—one that considers the potential risks and benefits of this powerful technology.

The European Union is currently drafting new regulations to better regulate AI systems. One of these regulations is the proposed AI Act [3]. This Act will introduce a risk-based approach to AI systems, which will be based on the principles of ethical AI. The principles of ethical AI include, but are not limited to, transparency, accountability, and human oversight. The AI Act will complement the General Data Protection Regulation (GDPR), which already partly regulates AI systems [9]. The GDPR provides rules on how personal data should be processed and how data subjects should be protected from solely automated decision-making. However, the GDPR does not fully address the ethical concerns that arise from the use of AI systems. The AI Act will, therefore, fill this gap by introducing additional rules and guidelines that will ensure ethical AI practices. Other parts of the world are also recognizing the need for such regulations. The US government has tasked the National Institute of Standards and Technology to develop standards and guidelines for AI implementation in health care [10] to ensure patient safety and privacy. In Asia, countries like China and Japan are developing their own regulations for the use of AI in health care, focusing on data privacy and algorithmic fairness. Similarly, in Africa, countries like South Africa and Kenya are working on regulations that address the ethical challenges of AI in health care, such as informed consent and safety.

A few governments, like the Singapore government, have issued good practice guidelines for developers and implementers of AI in health care [11], and regulatory organizations like the US FDA have issued guidelines for adaptive algorithms [4]. Beyond these very few examples, what is lacking in this discourse is the presence of precise or customized regulations for health care AI. We need to consider the specialized aspects of health care planning and delivery and the high impact any adversarial effects of health care AI can have on the individual or community’s well-being. Therefore, there is a need to consider a specialist pathway to regulate AI in health care. For instance, such a pathway could involve a framework for assessing the potential risks and benefits of health care AI as well as guidelines for the responsible deployment and ongoing monitoring of AI-driven health care solutions. In addition, it may be important to consider the roles of different stakeholders, including health care providers, patients, and regulatory bodies, in ensuring the ethical and effective use of AI in health care. By taking a proactive and holistic approach to regulating AI in health care, we can harness the potential of this technology to improve patient outcomes and advance public health objectives.

## The Need for Precise Regulation

Health care is a complex field that involves intricate ethical and medicolegal considerations. The World Health Organization has emphasized the importance of ethics and human rights in the design, deployment, and use of AI in health care [12]. This underscores the need for AI systems to be developed with a patient-centric approach, ensuring that the rights and interests of patients are prioritized over commercial interests. General regulations, while providing a broad framework, may not adequately address AI applications in health care. Precise regulations, on the other hand, can provide clear guidelines for each of these challenges, ensuring that AI is implemented ethically and legally. There are several arguments in favor of precise regulation. First, precise regulation can help to ensure that AI systems are safe and effective. By tailoring regulations to specific systems, regulators can identify and address potential risks. Second, precise regulation can help to promote innovation. By providing clear rules for developers, precise regulation can encourage them to develop new and innovative AI systems. Third, precise regulation can help to protect patients. By ensuring that AI systems are safe and effective, precise regulation can help to protect patients from harm.

One of the most significant challenges is ensuring the safety and quality of clinical AI applications [13,14]. With AI being used in critical areas of medicine, such as intensive and emergency care, it is important to ensure its safety and effectiveness. AI systems are highly complex and sensitive to changes in the environment, and their performance can decay over time. Therefore, it is crucial that these systems be continuously monitored and updated to ensure their long-term safety and effectiveness. Additionally, the issue of bias in AI systems is a significant challenge for health care [15]. AI systems are trained on data, and if these data are not diverse or representative, the AI system may not perform well for certain patient groups. For instance, systems trained primarily on data collected from individuals in high-income countries may not perform well for individuals in low- and middle-income settings. This highlights the need for AI systems to be carefully designed to reflect the diversity of socioeconomic and health care settings. Another challenge is medical liability. Who is responsible if something goes wrong when AI is administering clinical care? Is it the manufacturer, the user, or someone else? These are complex medicolegal questions that require careful consideration [2,15]. Patient data protection and privacy are also major concerns when it comes to health care AI. With the vast amounts of medical data that AI systems process, it is essential to ensure that these data are protected from unauthorized access and use. Lastly, the issue of transparency is crucial in building public trust in AI systems [16]. Manufacturers should have policies that ensure that data are clinically relevant, consistently acquired, and sufficiently transparent to support the trust and understanding of data users. This is particularly important given the potential for significant adverse events for patients, such as inaccurate diagnoses, discriminatory clinical practices, and private data leaks.

However, this is not to say general regulatory measures are without merit. First, general regulation can be more efficient. By developing a single set of regulations that applies to all AI systems, regulators can avoid the need to develop separate regulations for each new system or application. Second, general regulation can be more consistent. By applying the same rules to all AI systems, regulators can help to ensure that AI systems are treated fairly. Third, general regulation can be more flexible. General regulation can provide a foundation for precise regulation, while precise regulation can help to address specific risks and promote innovation. By allowing for flexibility in how the regulations are applied, regulators can adapt the regulations to changing circumstances.

## Developing Precise Regulation for AI in Health Care

Adapting general AI regulations to a more specific application in health care requires a nuanced understanding of both the broader context of AI and the unique characteristics of the health care sector. For example, adapting general AI regulations to health care necessitates a thorough understanding of the ethical principles that guide the sector, such as autonomy, beneficence, nonmaleficence, and justice [15,17]. This includes considering regulations that limit the use of patient data, which might be suitable in general AI applications but may need to be modified to allow for the use of deidentified patient data in AI-driven research in the health care sector [2,15]. General AI regulations often stress the importance of accountability and transparency, which are also crucial in the health care domain [15]. However, due to the sensitive nature of health-related data and the potential for adverse patient outcomes, these concepts need to be further emphasized. For instance, AI algorithms used for diagnosing or predicting health conditions should be fully explainable and their decision-making processes should be transparent for review and accountability [16].

General regulations, such as the GDPR, provide a framework for protecting personal data. However, health care data are particularly sensitive, and additional safeguards may be necessary to ensure that AI applications do not compromise patient privacy [2,15]. This could lead to stricter regulation and oversight of how AI systems in health care collect, store, and process patient data. For example, the regulation could mandate robust encryption standards, regular auditing of systems, and severe penalties for breaches. The Health Insurance Portability and Accountability Act (HIPAA) in the United States can be a key reference, as it requires that health care providers obtain patient consent before sharing their data. Similar regulations could be adopted to ensure that AI applications in health care are transparent about how they collect, store, and use patient data.

AI systems, in general, are required to undergo robust testing and validation. Regulations such as the European Union’s Medical Device Regulation provide a framework for ensuring the safety and efficacy of medical devices. However, AI applications in health care are not always classified as medical devices and may not be subject to the same regulations. To ensure that AI applications in health care are safe and effective, precise regulations could be developed that require AI applications to undergo rigorous testing and validation before they are deployed in clinical settings. In health care, this is especially important due to the high-stakes nature of many AI applications [1]. Regulations might need to specify more stringent testing standards, including comprehensive clinical trials and external validation across diverse populations, to ensure effectiveness and safety. Finally, adapting general AI regulations to health care would also mean integrating them with existing health care regulations, such as HIPAA in the United States or MyHealthRecord in Australia [18,19].

Adapting generic AI regulations for health care applications and facilitating their incorporation by AI vendors involves a series of steps and cooperative strategies. Regulatory agencies would first establish clear, specific guidelines adapted from generic AI regulations to cater to the unique needs and challenges of the health care sector [20]. These might encompass areas such as data privacy and security, algorithmic transparency and explainability, as well as patient rights. In developing these regulations, health agencies would engage with a variety of stakeholders, including AI vendors, health care providers, and patient advocacy groups. This collaboration ensures that the regulation is both practical and effective in its application [2]. To facilitate the adoption of these regulations by AI vendors, regulatory agencies might provide resources and support. This could include explanatory documents, training webinars, and consultation services [2,4].

**Figure 1:**
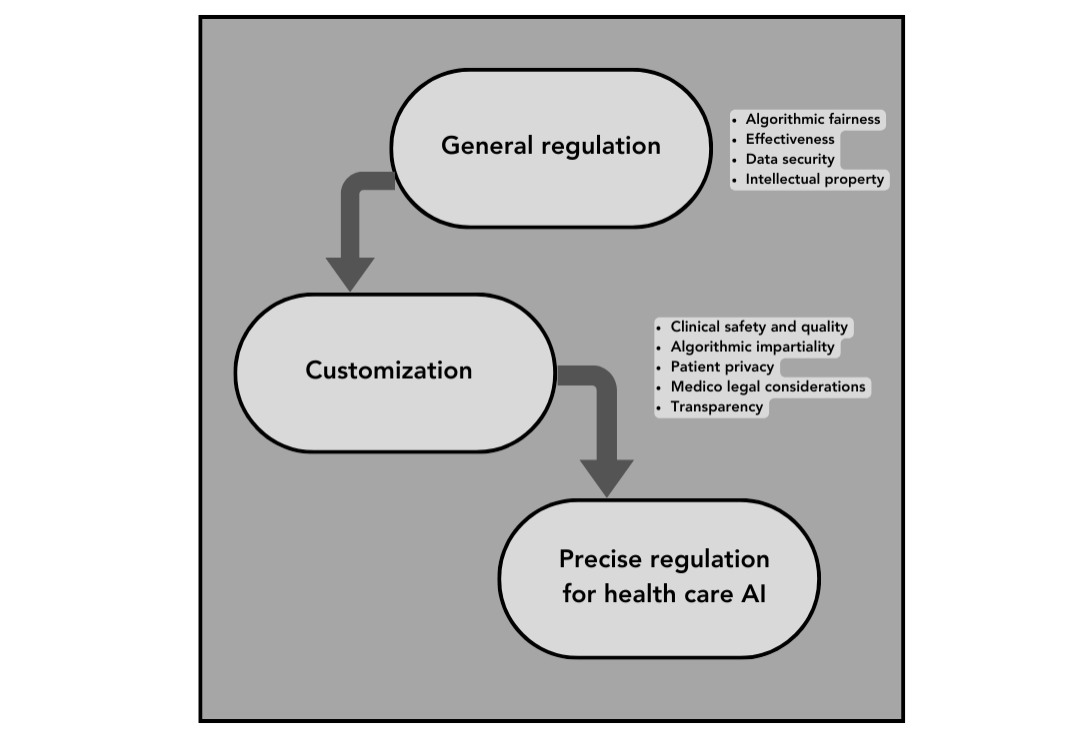
Precise Regulation. Customization of general regulation leads to better regulation of AI in healthcare.

Given the complexity and potentially disruptive nature of new regulations, agencies might opt for phased compliance. This would allow AI vendors to gradually implement changes, starting with the most critical areas, without abruptly disrupting their operations [4]. To ensure adherence to the regulations, agencies could set up regular audits or introduce a certification system. AI vendors who comply with the regulations and pass the audit could be awarded a certification, providing an incentive for compliance and a marker of trust for health care providers [4]. Post implementation, health agencies could establish a feedback mechanism for AI vendors. This would allow vendors to voice challenges or issues they are experiencing, and the agencies could use this feedback to refine and improve the regulations over time [21]. A real-world example of such adaptation is seen in the US FDA’s Proposed Regulatory Framework for Modifications to AI/Machine Learning-Based Software as a Medical Device [4]. This framework represents an attempt to balance the need for oversight with the unique characteristics of AI or machine learning applications and involves ongoing collaboration with vendors to ensure the practicality of the regulations (Figure 1).

## Discussion

In conclusion, the transformative potential of AI in health care is undeniable, but it also brings forth a myriad of ethical and legal challenges that require careful regulation. This paper has advocated for the necessity of precise regulation in the health care AI sector, rather than a general regulatory approach. Precise regulation, tailored to the specific needs and challenges of health care AI, can ensure the ethical and responsible use of this technology. It can address the unique complexities of health care, such as patient safety, data privacy, and medical liability, and can promote innovation by providing clear guidelines for AI developers. As AI continues to permeate health care, it is crucial that we develop and implement precise regulations that prioritize patient rights and interests while fostering the growth and development of AI technologies. This approach will ensure that AI serves as a tool for enhancing health care delivery, rather than a source of ethical and legal dilemmas.

## Abbreviations

AI: artificial intelligence
FDA: Food and Drug Administration
GDPR: General Data Protection Regulation
HIPAA: Health Insurance Portability and Accountability Act
